# Polycystin-1 Orchestrates Tumor Context-Dependent Mechanotransduction Programs Driving Epithelial-to-Mesenchymal Transition and Invasion in Solid Cancers

**DOI:** 10.7150/ijbs.131646

**Published:** 2026-03-25

**Authors:** Antonios N. Gargalionis, Kostas A. Papavassiliou, Angeliki-Ioanna Giannopoulou, Panagiotis Sarantis, Christina Piperi, Eleftheria Lakiotaki, Anastasios Stofas, Christos Adamopoulos, Panagiota Kontou, Efstathios Boviatsis, Stefanos Korfias, Nikolaos Kavantzas, Penelope Korkolopoulou, Efthimia K. Basdra, Athanasios G. Papavassiliou

**Affiliations:** 1 Department of Biological Chemistry, Medical School, National and Kapodistrian University of Athens, Athens 11527, Greece.; 2 First University Department of Respiratory Medicine, 'Sotiria' Chest Hospital, Medical School, National and Kapodistrian University of Athens, Athens 11527, Greece.; 3 First Department of Pathology, Medical School, National and Kapodistrian University of Athens, Athens 11527, Greece.; 4 Department of Mathematics, University of Thessaly, Lamia 35131, Greece.; 5 Second Department of Neurosurgery, 'Attikon' University Hospital, Medical School, National and Kapodistrian University of Athens, Athens 12642, Greece.; 6 First Department of Neurosurgery, 'Evaggelismos' General Hospital, Medical School, National and Kapodistrian University of Athens, Athens 10676, Greece.

**Keywords:** polycystin-1, polycystin-2, mechanotransduction, epithelial-to-mesenchymal transition (EMT), transcriptional coactivator with PDZ-binding motif (TAZ), solid malignancies

## Abstract

Mechanotransduction critically shapes tumor progression by regulating cytoskeletal remodeling, epithelial-to-mesenchymal transition (EMT), invasion, and immune evasion. Polycystin-1 (PC1) and polycystin-2 (PC2), established mechanosensitive proteins in epithelial systems, have recently been implicated in tumor biology; however, their roles across diverse solid malignancies remain insufficiently defined. We assessed PC1 and PC2 expression patterns and their associations with clinicopathological features in human lung, breast, prostate, and brain tumors. PC1 functional modulation was performed in xenograft models using an extracellular mechanosensitivity-blocking antibody, and in cancer cell lines via *polycystic kidney disease 1* (*PKD1*) siRNA. We evaluated consequences on EMT, tumor growth, migration, and the mechanotransduction effector TAZ. PC1 and PC2 exhibited strong positive correlation across multiple tumor types, indicating coordinated mechanobiological regulation in cancer. Their expression associated with clinically aggressive features, including PD-L1 expression in lung cancer, adverse pathological characteristics in prostate cancer, and poorer survival in HER2⁺ breast cancer with elevated PC2 levels. *In vivo*, inhibition of PC1 mechanosensing consistently attenuated EMT programs across tumor types, accompanied by reductions in tumor growth. *In vitro*, *PKD1* silencing reduced cell migration and induced context-dependent modulation of EMT markers. Notably, PC1 suppression induced TAZ activation in breast cancer and glioma cells, indicating a cell type-specific regulatory interaction between PC1 and Hippo-mechanotransduction signaling. Our data suggest that polycystins, PC1 in particular, exert conserved yet context-dependent mechanoregulatory functions in solid tumors. By influencing EMT, migration, tumor progression, and TAZ-mediated mechanotransduction, PC1 emerges as a potential biomarker and mechanotherapeutic target in mechanically responsive cancers.

## Introduction

Solid tumors exist within dynamic mechanical microenvironments that profoundly influence their biological behavior 1. Biophysical forces, ranging from extracellular matrix (ECM) stiffening and altered viscoelasticity to hydrostatic pressure and aberrant cell-cell or cell-matrix tension, are now recognized as potent regulators of tumor progression. These mechanical cues reshape transcriptional outputs through mechanoresponsive signaling pathways and drive hallmark oncogenic processes including epithelial-to-mesenchymal transition (EMT), invasion, metastasis, immune evasion, and therapeutic resistance 1-3. Central to these adaptations are mechanosensory proteins embedded within adhesive complexes and primary cilia, which translate external mechanical stimuli into intracellular biochemical signals 1, 2, 4. Among these, polycystin-1 (PC1) and polycystin-2 (PC2) are emerging as pivotal components of epithelial mechanotransduction. PC1, a large adhesion GPCR-like protein with a mechanosensitive extracellular domain, and PC2, a Ca²⁺-permeable ion channel, physically interact via their coiled coil domains to form a signaling complex positioned at the plasma membrane and primary cilia 5-10. Although mutations in *polycystic kidney disease 1* (*PKD1*) or *polycystic kidney disease 2* (*PKD2*) underlie autosomal dominant polycystic kidney disease (ADPKD), extensive work has revealed that polycystins regulate diverse pathways implicated in mechanobiology, including mechanistic target of rapamycin (mTOR), extracellular signal-regulated kinase (ERK), Janus kinase/signal transducers and activators of transcription (JAK/STAT), and Wingless-related integration site (Wnt) signaling, and modulate cytoskeletal dynamics, β-catenin stability, and transcriptional coactivator activity 5. In this respect, the polycystin complex is particularly attractive to study in cancer because it combines an adhesion GPCR-like mechanosensor (PC1) with an ion channel effector (PC2) within a cilia- and membrane-associated signaling module, thereby providing a mechanistically plausible bridge between extracellular forces, Ca²⁺-linked signaling, and transcriptional reprogramming. Their established roles in epithelial mechanosensing raise the possibility that polycystins contribute more broadly to cancer cell adaptation within mechanically altered TMEs 11.

Accumulating evidence supports this hypothesis 11-15. Previous work from our group has demonstrated that PC1 and PC2 expression correlates with aggressive clinical behavior in colorectal and renal cell carcinomas, and that PC1 overexpression enhances EMT and promotes invasive phenotypes in colorectal cancer models 12, 13. Conversely, we have also shown that PC1 inhibition suppresses EMT and alters mechanotransductive signaling in glioblastoma cells, particularly under conditions of elevated hydrostatic pressure 15. These studies suggest that the polycystin complex may function as a conserved mechanoresponsive module in tumor biology, yet its involvement across different solid tumor types remains largely unexplored. Importantly, it is unknown whether polycystins exert shared mechanobiological roles across cancers arising from tissues with distinct mechanical properties, or whether their effects are intrinsically context-dependent. The present work extends these prior tumor-specific observations by adopting a cross-tumor comparative design and integrating multi-cohort clinicopathological profiling across distinct solid malignancies with *in vivo* blockade of PC1 mechanosensitivity and mechanotransduction-effector readouts, enabling direct assessment of conserved versus context-dependent PC1 programs across different tumor lineages and mechanical milieus.

In the present study, we focused on lung, breast, prostate, and brain tumors because these malignancies develop within markedly distinct tissue architectures and biomechanical environments and collectively represent diverse modes of local invasion and therapeutic vulnerability, making them informative contexts in which to test whether polycystin-driven mechanobiological programs are conserved or tumor type-specific. By integrating clinicopathological analyses of human lung, breast, prostate, and brain tumors with functional modulation of PC1 *in vivo* and *in vitro*, we aimed to: (i) elucidate expression patterns and clinical correlations of PC1 and PC2 across tumor types, (ii) determine the impact of PC1 mechanosensitivity on EMT and tumor progression, (iii) characterize PC1-dependent effects on cancer cell migration, and (iv) assess how PC1 suppression influences the mechanotransduction effector TAZ. Collectively, this design is intended to define whether PC1/PC2 function as a shared mechanobiological module across solid cancers while revealing the degree to which downstream outputs, particularly EMT and TAZ-associated programs, diverge according to tumor lineage and baseline cell state. Our findings reveal a coordinated yet context-specific mechanoregulatory role for polycystins in solid tumor biology and highlight PC1 as a potential biomarker and therapeutic target in mechanically-driven cancer progression.

## Materials and Methods

### Cancer cell lines

MDA-MB-231 (triple-negative breast cancer; ATCC HTB-26), A549 (lung adenocarcinoma; ATCC CCL-185), PC3 (prostate adenocarcinoma; ATCC CRL-1435), and GOS-3 (astrocytic glioma; DSMZ ACC 408) cell lines were used. All cancer cell lines tested negative for mycoplasma contamination and were used within limited passage numbers to minimize phenotypic drift.

### Cell culture conditions

Cells were maintained at 37 ^°^C in a 5% CO_2_ incubator. Culture media were as follows: MDA-MB-231 and PC3 were cultured in high-glucose Dulbecco's Modified Eagle Medium (DMEM) supplemented with 10% fetal bovine serum (FBS) and 1% penicillin-streptomycin (10,000 U/mL penicillin-10,000 mg/mL streptomycin), GOS-3 were cultured in RPMI-1640 medium with stable L-glutamine supplemented with 10% FBS and 1% penicillin-streptomycin, and A549 were cultured in Ham's F-12 medium with stable L-glutamine supplemented with 10% FBS and 1% penicillin-streptomycin. All reagents were used at manufacturer-recommended concentrations, and cell morphology and confluence were monitored by phase-contrast microscopy.

### *PKD1* knockdown by small interfering RNA (siRNA)

*PKD1* was silenced using ON-TARGETplus SMARTpool siRNA (Dharmacon, L-007666-00-0005). A non-targeting (nt) siRNA (Dharmacon D-001210-01-05) served as control. Cells were seeded in 12-well plates to achieve ~70-90% confluency at transfection. Transfection was performed using Lipofectamine 3000 (Invitrogen) according to the manufacturer's protocol, at final concentrations of 20 nM (*PKD1* siRNA) and 1 nM (control, nt siRNA). After 5-6 h, complete medium was added. Cells were harvested 24 h post-transfection for downstream analyses. Knockdown efficiency was confirmed by Western blotting.

### Wound healing assay

Cells were seeded at a density of 2-3 × 10^5^ cells per well (density adjusted by cell line) on 12-well plates. A sterile 200-µL pipette tip was used to create a scratch to the cell monolayer throughout the well and cells were either transfected with *PKD1* or nt siRNAs. Images of the same wound region were acquired at 0, 24, and 48 h using a phase-contrast microscope at 20× magnification. Wound closure was quantified using ImageJ software and the wound healing size tool plugin. Migration was expressed as the percentage of wound area covered relative to 0 h.

### Protein extraction and western blotting

Cells were collected in ice-cold phosphate-buffered saline (PBS) with a cell scraper from 12-well plates 24 h post-transfection. To collect the cell pellets, a centrifugation step followed at 5000 rpm for 5 min and the supernatant was discarded. Protein extraction from cell lines was performed using ice-cold Cell Lysis Buffer (Cell Signaling Technology #9803) supplemented with Protease/Phosphatase Inhibitor Cocktail (Cell Signaling Technology #5872). Proteins were resolved by electrophoresis in 8-10% sodium dodecyl sulfate-polyacrylamide gel electrophoresis (SDS-PAGE) gels, transferred to nitrocellulose membranes (Porablot NCP, Macherey-Nagel, Germany, #12807411), and blocked in Tris-buffered saline Tween-20 (TBS-T 0.1%) with 5% non-fat milk and 0.02% NaN_3_). Membranes were incubated overnight at 4 ^°^C with the following primary antibodies: PC1 (2741 PC1-CT; 1:1000 dilution, kindly provided by the Nephrology Department of Medicine, University of Maryland Baltimore), β-catenin (Santa Cruz Biotechnology, sc-59737; 1:400 dilution), Vimentin (Santa Cruz Biotechnology, sc-73259; 1:400 dilution), TAZ (Cell Signaling Technology #8418; 1:1000 dilution), and β-actin (Santa Cruz Biotechnology, sc-47778; 1:1000 dilution). After incubation with horseradish peroxidase (HRP)-conjugated secondary antibodies (anti-Mouse IgG, Cell Signaling Technology #7076, 1:9000 dilution; anti-Rabbit IgG, Cell Signaling Technology #7074, 1:5000 dilution), immunoreactive bands were detected with the SuperSignal™ West Pico PLUS Chemiluminescent Substrate (Thermo Fisher Scientific, #34580). Relative proteins amounts were evaluated by densitometry using ImageJ software and normalized to β-actin (loading control). Each experiment was performed at least three times and representative results are shown.

### Human tumor xenografts

NOD scid gamma (NSG) mice, aged 6-8 weeks, were injected subcutaneously in the flank region with 3 × 10^6^ to 6 × 10^6^ human cancer cells (A549, PC3, MDA-MB-436, or GOS-3) suspended in complete growth medium (150-200 μL). Once tumors became palpable, mice received daily subcutaneous injections of 50 μL PC1 inhibitory antibody (IgPC1) for 10 consecutive days, while control mice received daily subcutaneous injections of 50 μL IgG for 10 consecutive days. At the end of treatment, tumors were excised post-euthanasia, weighed, measured, and processed for immunohistochemical analysis.

### Immunohistochemistry (IHC)

Formalin-fixed, paraffin-embedded (FFPE) tissue sections of breast (n = 90), lung (n = 54), prostate (n = 99), and glioma (n = 87) tumors were processed using standard protocols. Sections (4-μm thick) were deparaffinized by incubation at 62^o^C (30 min), washed three times consecutively in xylene (3 min each), rehydrated through different ethanol gradients (100%, 96%, 80%, and 70%), and rinsed with distilled water. Antigen retrieval was carried out by heating the sections in citrate buffer (pH 6.0) at 95 ^°^C for 20 min. Endogenous peroxidase activity was quenched by incubating the samples in 3% H_2_O_2_ for 10 min at room temperature. After washing with PBS, non-specific binding was blocked with 5% normal goat serum (NGS) for 1 h. Sections were then incubated overnight at 4 ^°^C with primary antibodies (PC1, sc-10371; PC2, sc-47734; E-cadherin, sc-8426; Vimentin, sc-73259; all diluted 1:100 in blocking buffer). Following PBS washes, biotinylated secondary antibody (Merck Millipore, cat. # 20775) and Streptavidin-HRP conjugate (Merck Millipore, cat. # 20774) were applied sequentially (10 min each). 3,3′-diaminobenzidine (DAB) was used for immunoreactivity visualization, and nuclei were counterstained with hematoxylin (Sigma-Aldrich, St. Louis, MO, USA). Sections were dehydrated through increasing ethanol concentrations (70%, 80%, 96%, and 100%) and mounted with a permanent mounting medium onto glass coverslips. Staining was quantified using the Histo-score (H-score) method, calculated as intensity × percentage of positive tumor cells.

### Statistical analysis

Analyses and graphical representations were performed using SPSS v25 (IBM Corp., Armonk, NY, USA) and GraphPad Prism v5 (GraphPad Software, San Diego, CA, USA). Bivariate Pearson, Spearman, and Phi coefficients were used for correlation analyses. Comparisons between groups were assessed via two-sided Student's *t*-test, ANOVA, Mann-Whitney U-test, and Kruskal-Wallis H-test, as appropriate. Survival outcomes were evaluated using Kaplan-Meier analysis and differences between survival curves were assessed using the log-rank test. Multinomial logistic regression analysis was chosen for its interpretability and ability to model categorical outcomes 16. A two-sided *p*-value ≤ 0.05 was considered indicative of statistical significance throughout all analyses.

## Results

### Coordinated expression of polycystins across solid tumors and clinicopathological correlations

**Figure 1:**
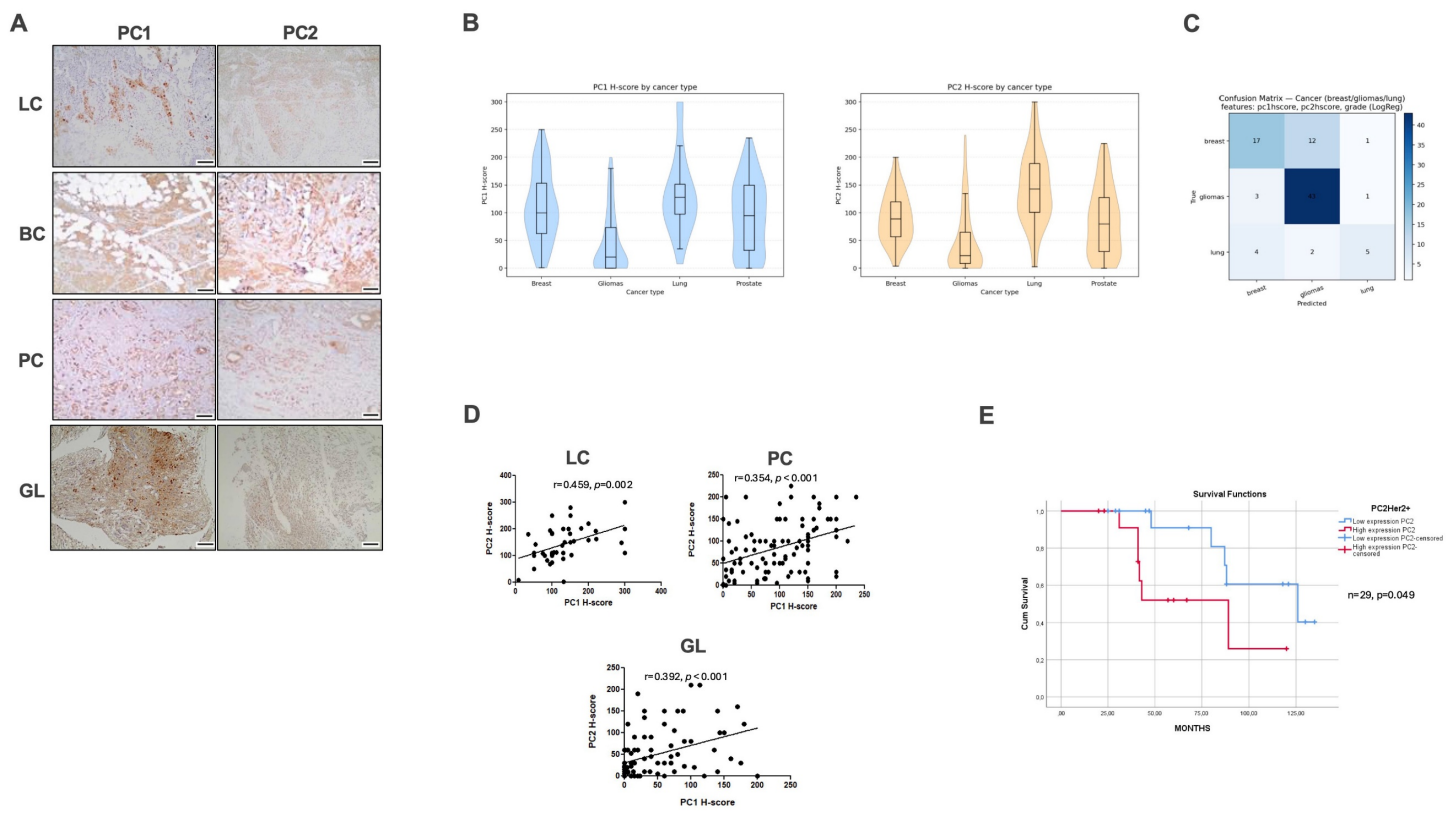
**Coordinated expression of polycystins across solid tumors and clinical correlations. (A)** Representative immunohistochemical staining of PC1 and PC2 in human lung cancer (LC), breast cancer (BC), prostate cancer (PC), and glioma (GL) specimens. **(B)** Violin plots showing distribution of PC1 and PC2 H-scores across tumor types, with boxplots indicating median and interquartile range. Kruskal-Wallis testing revealed significant differences in PC1 and PC2 expression across cancer types. **(C)** The confusion matrix summarizes the performance of a multinomial logistic regression classifier that attempted to predict tumor type (breast, lung, glioma, prostate) using tumor grade, PC1 H-score, and PC2 H-score. Each row of the matrix represents the true tumor type, while each column represents the model-predicted tumor type (correct classifications lie on the diagonal; misclassifications are shown in the off-diagonal cells).** (D)** Scatter plots illustrating positive correlations between PC1 and PC2 H-scores in LC, PC, and GL. Pearson correlation coefficients and corresponding *p*-values are indicated. **(E)** Kaplan-Meier survival curves for HER2^+^ breast cancer patients stratified by high versus low PC2 expression, demonstrating prolonged survival in the low-PC2 group (log-rank test). Scale bars = 50 μm. HER2^+^, human epidermal growth factor receptor 2-positive; PC1, polycystin-1; PC2, polycystin-2.

To investigate whether polycystins contribute broadly to the biology of solid malignancies, we initially evaluated PC1 and PC2 expression in human lung, breast, prostate, and brain tumors (Figure 1A). Both proteins exhibited significant, tumor-type-specific variability. Kruskal-Wallis testing confirmed differences in PC1 and PC2 H-scores across tissues (PC1: H = 66.09, *p* < 0.0001; PC2: H = 65.38, *p* < 0.0001), with gliomas showing the lowest expression levels overall (Figure 1B). Multinomial logistic regression using tumor grade, PC1 H-score, and PC2 H-score (confusion matrix) showed limited discriminative power across all four malignancies (accuracy 56.6%). Excluding prostate cancer improved performance to 73.9% (Figure 1C). According to our analysis, gliomas are predicted accurately (43/47 correctly classified), while breast and lung tumors exhibited variable classification performance. Prostate cancer showed zero recall and was excluded from subsequent model refinement. Across lung, prostate, and glioma samples, PC1 and PC2 levels were strongly positively correlated (lung: r = 0.459, *p* = 0.002; prostate: r = 0.354, *p* < 0.001; gliomas: r = 0.392, *p* < 0.001) (Figure 1D).

To further characterize associations with clinicopathological parameters, we examined correlations within each tumor type. In lung cancer, both PC1 and PC2 exhibited negative correlations with the proliferation marker Ki-67 (PC1: r = -0.508, *p* = 0.053; PC2: r = -0.571, *p* = 0.026). PC1 positively correlated with programmed death-ligand 1 (PD-L1) expression (r = 0.374, *p* = 0.013) and with pembrolizumab-treated patients (r = 0.345, *p* = 0.023). A weak positive trend was observed between PC1 and epidermal growth factor receptor (EGFR)-mutant status (r = 0.285, *p* = 0.067). In breast cancer, human epidermal growth factor receptor 2-positive (HER2^+^) tumors displayed lower PC1 expression than human epidermal growth factor receptor 2-negative (HER2^-^) tumors (mean 98.02 vs 130). PC2 correlated with progesterone receptor status in a HER2-dependent manner (HER2^+^: r = -0.372, *p* = 0.051; HER2^-^: r = 0.595, *p* = 0.015). Importantly, lower PC2 expression was associated with longer survival in HER2^+^ patients (Figure 1E). In prostate cancer, PC1 correlated with extraprostatic extension (r = 0.293, *p* = 0.003) and tumor stage (r = 0.237, *p* = 0.019), while PC2 correlated with excision margin positivity (r = 0.222, *p* = 0.027), extraprostatic extension (r = 0.212, *p* = 0.035), seminal vesicle invasion (r = 0.210, *p* = 0.037), and advanced stage (r = 0.200, *p* = 0.048). Finally, in gliomas, PC2 expression was significantly higher in high-grade tumors (grades 3-4) than in grade 2 tumors (*p* = 0.007). In addition, both PC1 and PC2 localized preferentially to peri-necrotic and peri-vascular regions.

### Inhibition of PC1 mechanosensitivity suppresses EMT in human tumor xenografts

**Figure 2:**
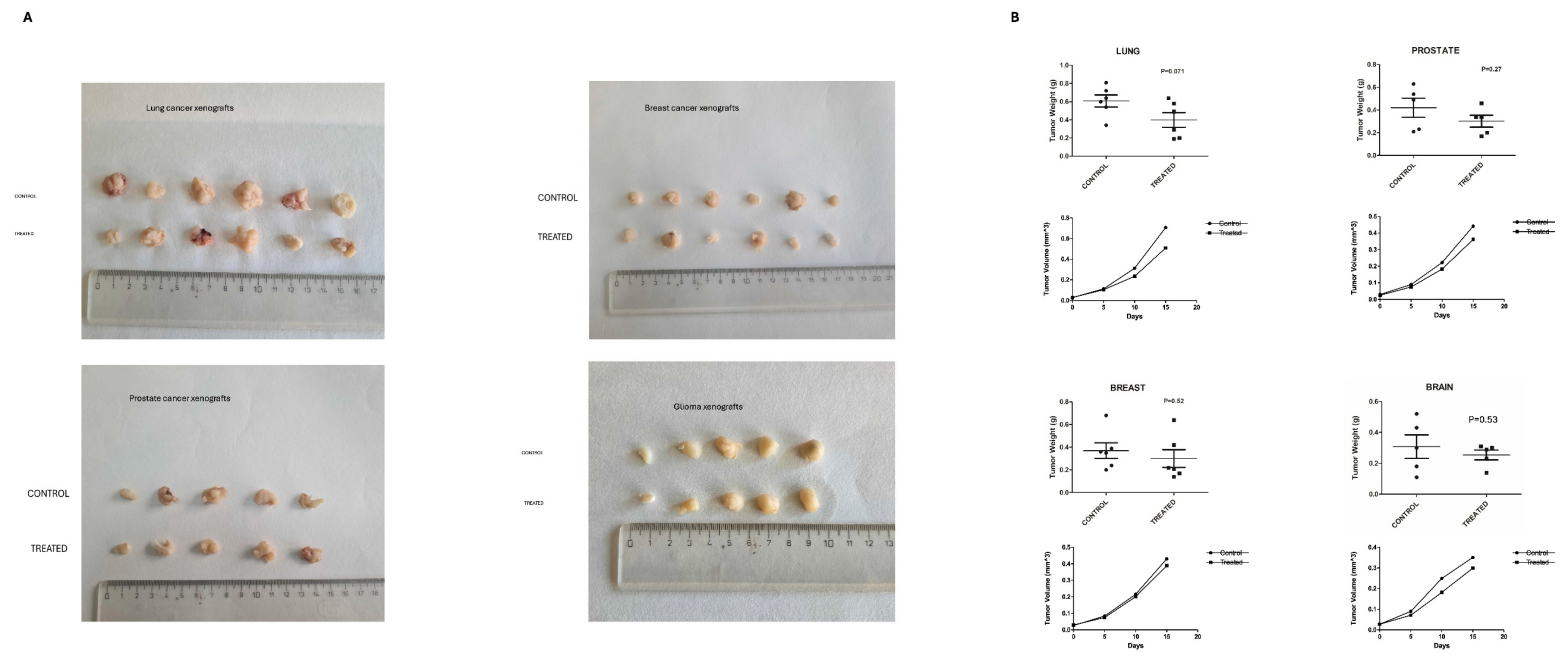
**Blockade of PC1 mechanosensitivity suppresses tumor growth in human tumor xenografts. (A)** Representative gross images of excised lung, prostate, breast, and glioma xenograft tumors from control (IgG-treated) and treated (IgPC1-treated) mice are shown, illustrating tumor size and morphological differences. **(B)** Quantification of tumor volume and weight change in lung, breast, prostate, and brain (glioma) xenografts following treatment with IgPC1 (treated) or IgG (control). Bar graphs show mean ± SEM. Statistical significance was assessed using Student's *t*-test and exact *p*-values are indicated on the plots.

**Figure 3:**
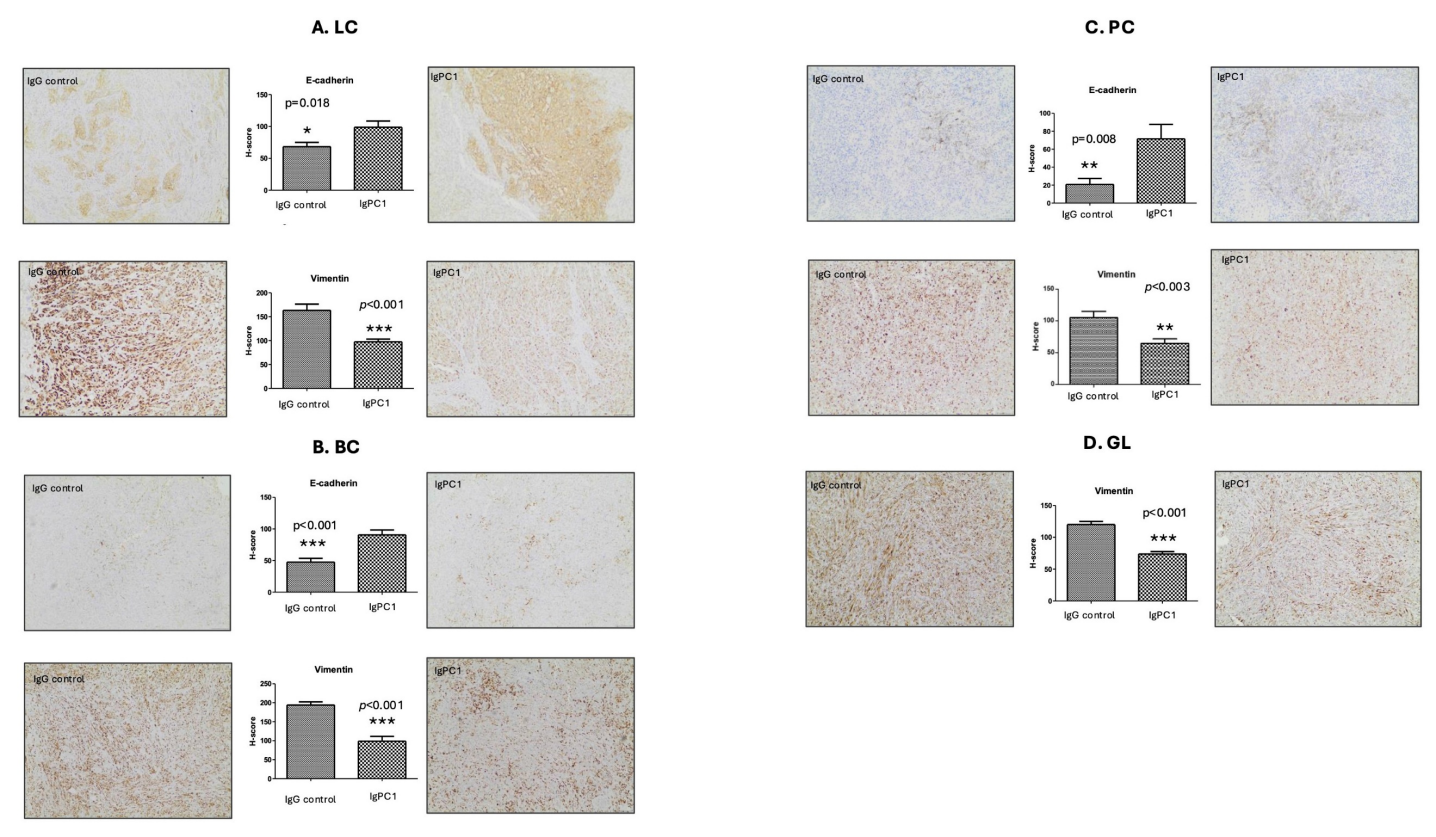
**Inhibition of PC1 mechanosensitivity suppresses EMT in human tumor xenografts.** Representative immunohistochemical staining and H-score quantification of E-cadherin (epithelial marker) and vimentin (mesenchymal marker) in xenografts derived from **(A)** lung cancer (LC), **(B)** breast cancer (BC), **(C)** prostate cancer (PC), and **(D)** glioma (GL) cell lines, treated with IgPC1 or control IgG. Bar graphs represent mean ± SEM. Statistical significance was assessed using Student's *t*-test (*p < 0.05; **p < 0.01; ***p < 0.001). Exact *p*-values for all statistically significant comparisons are provided in the bar graphs. Scale bars = 50 μm. EMT, epithelial-to-mesenchymal transition; PC1, polycystin-1.

To determine the functional role of PC1 in tumor progression, xenografts derived from lung (A549), prostate (PC3), breast (MDA-MB-436), and glioma (GOS-3) cells were treated with an antibody blocking the extracellular N-terminal mechanosensory domain of PC1 (IgPC1). Across all tumor types, PC1 inhibition resulted in modest but consistent reductions in tumor volume and weight**,** ranging from 9.6% (breast) to 28.0% (lung; *p* = 0.071) (Figure 2A-B). Alterations in the expression of EMT-associated proteins were robust and uniform across tumor xenograft models. The expression of E-cadherin, an epithelial marker, increased significantly in lung (*p* = 0.018), breast (*p* < 0.001), and prostate tumors (*p* = 0.008) (Figure 3A-C), whereas the expression of vimentin, a mesenchymal marker, decreased significantly in all four tumor types (lung, *p* < 0.001; breast, *p* < 0.001; prostate, *p* < 0.003; brain, *p* < 0.001) (Figure 3A-D).

### *PKD1* silencing reduces migration in cancer cell lines

**Figure 4:**
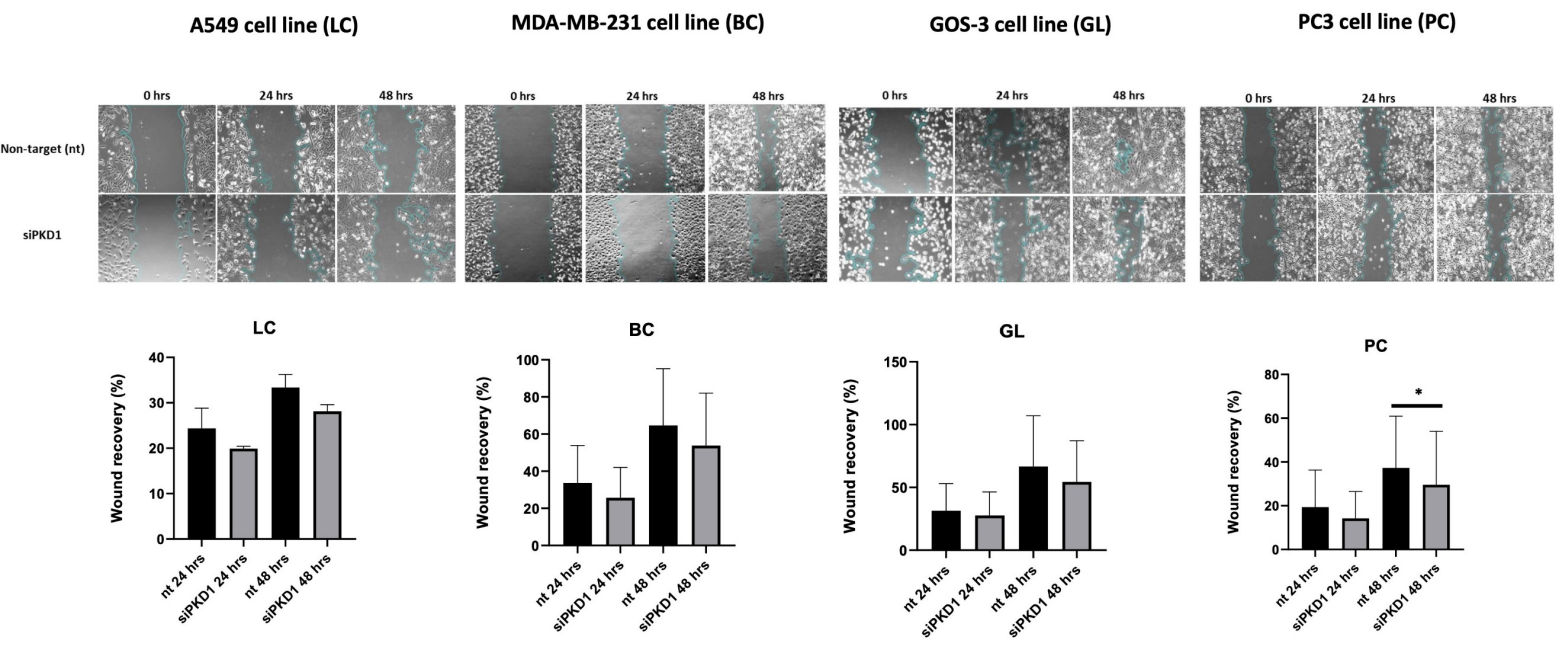
***PKD1* silencing reduces migration in cancer cell lines.** Representative wound-healing assay images and corresponding quantification of wound recovery at 0, 24, and 48 h in A549 (lung cancer, LC), MDA-MB-231 (breast cancer, BC), GOS-3 (glioma, GL), and PC3 (prostate cancer, PC) cells transfected with non-targeting (nt) siRNA or *PKD1*-specific siRNA (siPKD1). Bars represent mean ± SD from at least three independent experiments. Statistical significance was assessed using Student's *t*-test (*p < 0.05). Exact *p*-values for all statistically significant comparisons are provided in the bar graphs. Scale bars = 100 μm. Magnification used for wound-healing assay images: 20×. PKD1, polycystic kidney disease 1; siRNA, small interfering RNA.

We next evaluated the role of PC1 in cell motility using wound healing assays. Across all cancer cell lines tested, *PKD1* knockdown via siRNA resulted in reduced wound closure at both 24 and 48 h compared with non-targeting (nt) controls (Figure 4). Temporal analysis revealed that inhibition of wound closure was already detectable at 24 h and, in most cell lines, persisted or increased at 48 h. Specifically, for A549 cells, wound recovery was decreased by 4.5% at 24 h and by 5% at 48 h; for MDA-MB-231 cells, wound recovery was reduced by 8% at 24 h and by 11% at 48 h; for GOS-3 cells, wound recovery declined by 3.8% at 24 h and by 12% at 48 h; and for PC3 cells, wound recovery diminished by 5% at 24 h and by 8% at 48 h. These time-dependent measurements indicate sustained reduction in migratory capacity following PKD1 silencing.

### Context-dependent regulation of EMT markers following *PKD1* silencing

**Figure 5:**
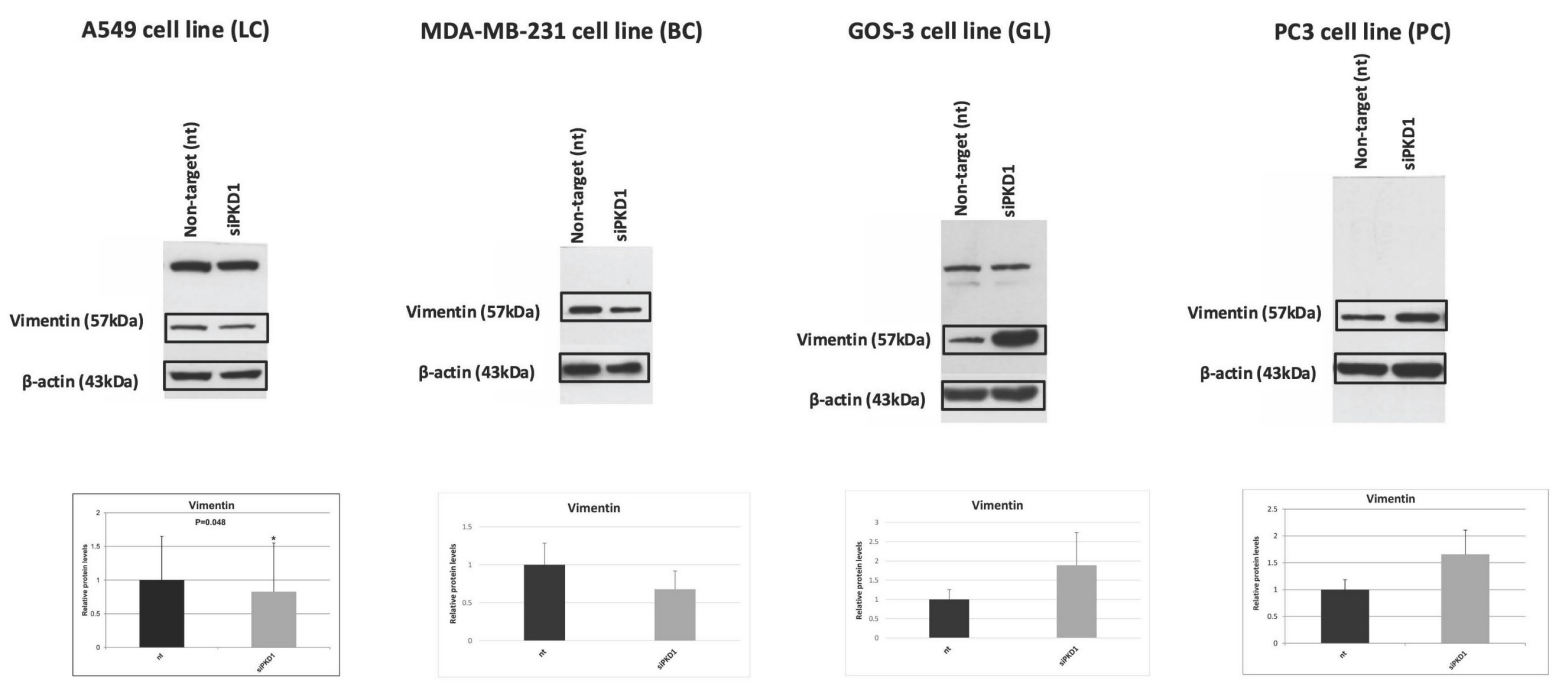
**Context-dependent regulation of the EMT marker vimentin following *PKD1* silencing.** Western blot analysis and densitometric quantification of vimentin in A549 (lung cancer, LC), MDA-MB-231 (breast cancer, BC), GOS-3 (glioma, GL), and PC3 (prostate cancer, PC) cells transfected with non-targeting (nt) siRNA or *PKD1*-specific siRNA (siPKD1). β-actin served as loading control. Bars represent mean ± SD of three independent experiments. Statistical significance was assessed using Student's *t*-test (*p < 0.05). Exact *p*-values for all statistically significant comparisons are provided in the bar graphs. β-actin bands were derived from gels that were run in parallel under exactly the same conditions, electrotransferred, and the corresponding nitrocellulose membranes were probed with the target protein. Each experiment was performed at least three times and representative results are shown. EMT, epithelial-to-mesenchymal transition; PKD1, polycystic kidney disease 1; siRNA, small interfering RNA.

**Figure 6:**
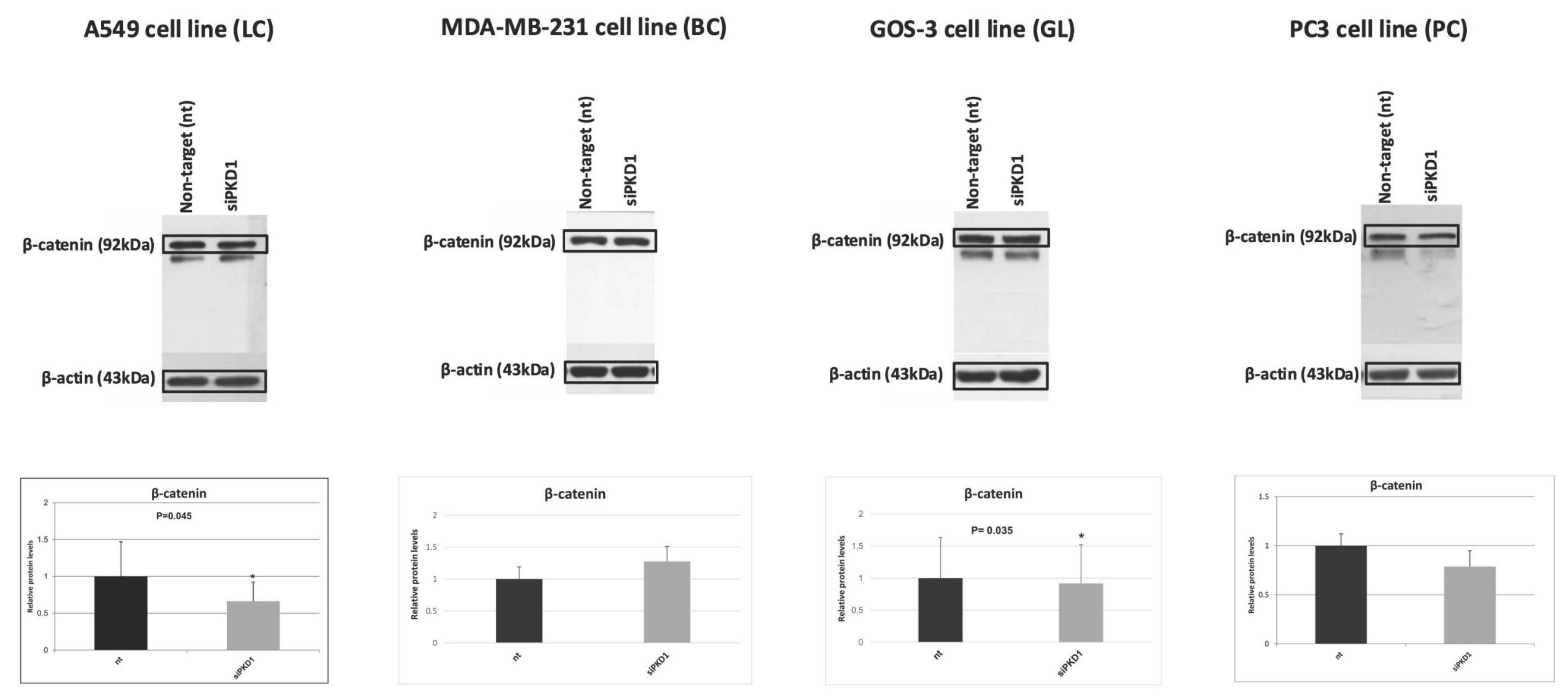
**Context-dependent regulation of the EMT marker β-catenin following *PKD1* silencing.** Western blot analysis and densitometric quantification of β-catenin in A549 (lung cancer, LC), MDA-MB-231 (breast cancer, BC), GOS-3 (glioma, GL), and PC3 (prostate cancer, PC) cells transfected with non-targeting (nt) siRNA or *PKD1*-specific siRNA (siPKD1). β-actin served as loading control. Bars represent mean ± SD of three independent experiments. Statistical significance was assessed using Student's *t*-test (*p < 0.05). Exact *p*-values for all statistically significant comparisons are provided in the bar graphs. β-actin bands were derived from gels that were run in parallel under exactly the same conditions, electrotransferred, and the corresponding nitrocellulose membranes were probed with the target protein. Each experiment was performed at least three times and representative results are shown. EMT, epithelial-to-mesenchymal transition; PKD1, polycystic kidney disease 1; siRNA, small interfering RNA.

We next examined EMT markers, including vimentin (Figure 5) and β-catenin (Figure 6), in cancer cell lines following PKD1 knockdown via siRNA. Lung cancer cells (A549) displayed decreased vimentin (0.8-fold) and β-catenin (0.7-fold) compared with non-target controls. Breast cancer cells (MDA-MB-231) exhibited decreased vimentin (0.7-fold) but increased β-catenin (1.3-fold). Glioma cells (GOS-3) demonstrated increased vimentin (1.9-fold) with minimal change in β-catenin (0.9-fold), while prostate cancer cells (PC3) presented increased vimentin (1.9-fold) and decreased β-catenin (0.8-fold).

### *PKD1* silencing induces TAZ activation in a tumor type-dependent manner

**Figure 7:**
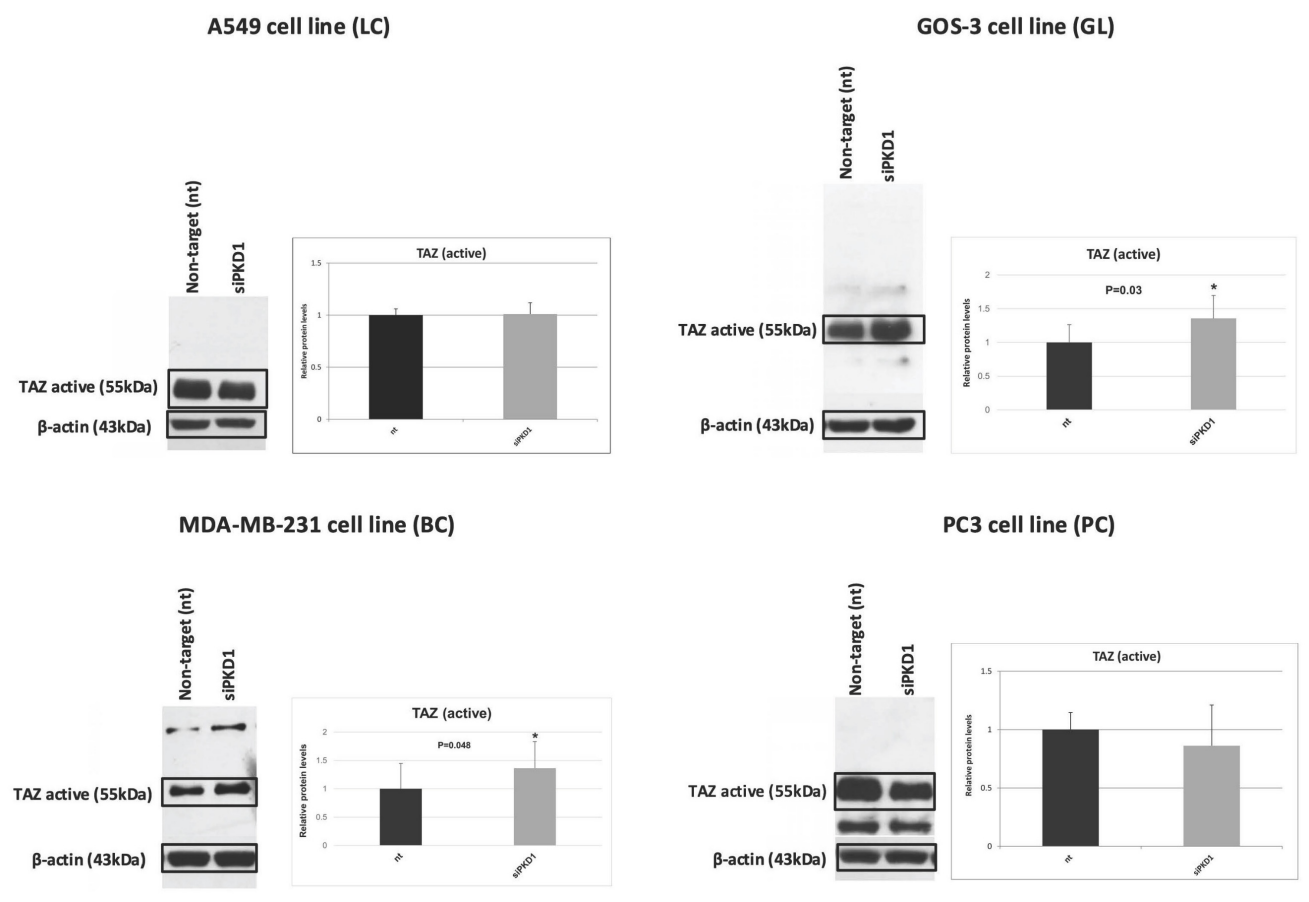
***PKD1* silencing induces TAZ activation in a tumor-type-dependent manner.** Western blot analysis and quantification of non-phosphorylated (active) TAZ in A549 (lung cancer, LC), MDA-MB-231 (breast cancer, BC), GOS-3 (glioma, GL), and PC3 (prostate cancer, PC) cells following non-targeting (nt) siRNA or *PKD1*-specific siRNA (siPKD1) transfection. β-actin was used as loading control. Bars represent mean ± SD of three independent experiments. Statistical significance was assessed using Student's *t*-test (*p < 0.05). Exact *p*-values for all statistically significant comparisons are provided in the bar graphs. β-actin bands were derived from gels that were run in parallel under exactly the same conditions, electrotransferred, and the corresponding nitrocellulose membranes were probed with the target protein. Each experiment was performed at least three times and representative results are shown. PKD1, polycystic kidney disease 1; siRNA, small interfering RNA; TAZ, transcriptional coactivator with PDZ-binding motif.

To assess PC1's role in mechanotransduction, we measured unphosphorylated (active) TAZ after *PKD1* silencing (Figure 7). Active TAZ remained unchanged in A549 lung cancer (1.0-fold) and PC3 prostate cancer (0.9-fold) cells. Conversely, active TAZ increased significantly in MDA-MB-231 breast cancer and GOS-3 glioma cells (1.4-fold each; *p* = 0.05 and *p* = 0.03).

Collectively, our results demonstrate coordinated expression of PC1 and PC2 across multiple solid tumors with tumor-type-specific clinicopathological associations. Functional inhibition of PC1 mechanosensitivity *in vivo* was associated with consistent modulation of EMT markers across xenograft models, while PKD1 silencing *in vitro* reduced migratory capacity across cancer cell lines and induced cell type-dependent changes in EMT-associated proteins and TAZ activation. These observations provide a multi-level characterization of PC1-related effects across distinct tumor contexts.

## Discussion

Mechanical forces are now recognized as central regulators of tumor initiation, progression, and therapeutic response, acting through complex mechanotransduction pathways that convert physical cues into transcriptional and phenotypic outputs in cancer cells 3, 17-24. Among the molecular systems proposed to participate in these processes, the polycystin family, particularly PC1 and PC2, has emerged as a mechanosensory module with diagnostic and prognostic potential in cancer 11. Early studies from our group demonstrated that PC1 and PC2 are involved in the acquisition of aggressive phenotypes in colorectal cancer 12, while subsequent work revealed that PC1 promotes angiogenesis and activates the phosphoinositide 3-kinase (PI3K)/protein kinase B (AKT)/mTOR pathway in renal cell carcinoma 13 and modulates cancer cell behavior through interactions with mTOR and JAK signaling cascades 14.

Additional prior work from our group has shown that in glioblastoma PC1 cooperates with hydrostatic pressure to drive pathogenic processes *in vitro* 15, reinforcing the concept that polycystins integrate mechanical cues with oncogenic signaling. At the mechanistic level, PC1 undergoes regulated cleavage and nuclear translocation events that influence transcriptional programs 25, potentiates differentiation pathways through JAK2/STAT3 signaling 26, and regulates STAT activity via dual mechanisms 27. Polycystin signaling is also linked to Hippo pathway modulation, as altered Hippo activity has been documented in ADPKD 28 and PC1 has been shown to regulate bone development through direct interaction with the transcriptional coactivator TAZ 29, providing a direct conceptual bridge between polycystins and canonical mechanotransduction effectors. Finally, PC1 has been reported to regulate cell migration through PI3K-dependent cytoskeletal rearrangements and glycogen synthase kinase-3 beta** (**GSK3β)-mediated mechanical adhesion 30, further supporting its role in force-responsive cellular behaviors. Together, this body of work laid the conceptual foundation for the present study, in which we investigate whether polycystins function as mechanobiological regulators across multiple solid tumor types in clinically relevant settings.

In this study, we identify the polycystin complex, particularly PC1, as a tumor context-dependent regulator of mechanotransduction programs across multiple solid malignancies. By integrating human tissue IHC, xenograft modulation of PC1 mechanosensing, and *in vitro* analyses of migration and Hippo pathway effector activation, we show that polycystins form coordinated expression signatures associated with clinically relevant features, modulate epithelial-mesenchymal plasticity *in vivo*, and selectively restrain the activity of the mechanotransduction effector TAZ *in vitro*. Together, these findings position PC1 as a mechanobiological node linking extracellular physical cues to invasion-associated transcriptional programs in cancer.

Our IHC analyses across lung, breast, prostate, and brain tumors reveal that polycystins are not uniformly expressed but instead display tumor type-specific expression patterns that associate with clinically meaningful features, underscoring their functional relevance in cancer mechanobiology. The strong positive correlations between PC1 and PC2 in lung, prostate, and glioma tissues support the existence of a coordinated polycystin signaling module in solid tumors, consistent with their established biochemical interactions as a mechanosensory complex 31. In line with this, multinomial logistic regression using tumor grade together with PC1 and PC2 expression accurately classified gliomas but not prostate tumors, indicating that polycystin-associated mechanobiological signatures are highly tumor-context dependent rather than universal across solid malignancies. This resonates with previous findings suggesting that the role of contextual mechanobiological signaling in defining tumor phenotypes is increasingly recognized as critical for interpreting heterogeneous cancer behaviors 32-35.

Notably, PC1 expression correlated with PD-L1 levels and immunotherapy-treated status in lung cancer, suggesting that mechanosensory signaling may intersect with immune-modulatory programs in mechanically-stressed TMEs 36-39. This observation is further supported by emerging evidence that tumor mechanics can regulate immune-checkpoint programs through canonical mechanotransduction hubs. For example, matrix stiffening has been shown to increase PD-L1 expression via YAP-dependent mechanisms in lung adenocarcinoma models, linking mechanical cues to immune evasion pathways 38. In addition, recent work has highlighted broader Hippo-YAP/TAZ roles in immune escape phenotypes (including macrophage-related immune evasion programs), underscoring the plausibility that polycystin-driven mechanosensing may converge on immunoregulatory outputs in specific contexts 40. In prostate cancer, associations between polycystin expression and extraprostatic extension, seminal vesicle invasion, margin positivity, and tumor stage point to a role for PC1 and PC2 in local invasion and tissue remodeling, processes inherently driven by biomechanical forces 41. In breast cancer, the association between low PC2 expression and improved overall survival in HER2^+^ disease implies that polycystins may integrate with receptor-driven oncogenic circuits to influence tumor aggressiveness. Correspondingly, HER2 has been reported to detect extracellular mechanical signals and allow cancer cells to respond by enhancing actomyosin-dependent contractions and cell spreading on rigid surfaces 42. Finally, the enrichment of PC1 and PC2 in peri-necrotic and peri-vascular glioma niches, regions characterized by hypoxia, interstitial pressure, and altered stiffness, provide spatial evidence that polycystins are preferentially engaged in mechanically extreme microenvironments.

Functionally, blockade of PC1 mechanosensitivity with IgPC1 in xenografts produced a uniform epithelial shift across lung, breast, prostate, and glioma mouse models, as reflected by increased E-cadherin and decreased vimentin expression. These findings indicate that PC1 mechanosensing contributes to the maintenance of mesenchymal traits in the intact TME. The consistency of this EMT attenuation, despite only modest effects on tumor growth, suggests that PC1 primarily modulates tumor cell state and invasive potential rather than bulk proliferative capacity, aligning with the emerging concept of EMT as a driver of phenotypic plasticity rather than simple growth control 43, 44.

In contrast to the uniform *in vivo* EMT phenotype, *PKD1* silencing *in vitro* induced tumor context-dependent remodeling of EMT-associated proteins. The most epithelial-like cancer cell model, A549 lung cancer cells, showed reductions in both vimentin and β-catenin, whereas mesenchymal-like and non-epithelial models displayed heterogeneous EMT marker changes. These differences likely reflect the distinct nature of the two perturbations; IgPC1 selectively disrupts extracellular mechanosensing while preserving PC1 expression and adhesion-complex integrity, whereas *PKD1* siRNA reduces total PC1 abundance and distorts both structural and signaling functions in the mechanically simplified environment of 2D culture. Despite this EMT marker heterogeneity, *PKD1* silencing consistently reduced migration across all cancer cell lines, providing a functional correlate that bridges the *in vivo* and *in vitro* datasets. This uniform impairment of migratory capacity supports a role for PC1 in promoting invasion-related phenotypes, with EMT marker-level differences reflecting baseline epithelial-mesenchymal state and lineage-specific wiring rather than experimental inconsistency.

A central question in mechanobiology is how extracellular forces are translated into nuclear transcriptional programs that promote tumor progression. The activity of the transcriptional coactivator TAZ is widely accepted as a direct readout of mechanotransductive signaling, with its phosphorylation state determining nuclear localization and function 45-47. In our study, the selective induction of unphosphorylated (active) TAZ following *PKD1* silencing in breast cancer and glioma cells provides further mechanistic insight. As unphosphorylated TAZ represents the transcriptionally competent Hippo pathway effector, these findings indicate that PC1 normally restrains TAZ activity in specific tumor contexts. The absence of this response in lung and prostate cancer cells suggests that PC1 does not act as a universal upstream regulator of Hippo signaling, but rather fine-tunes force-responsive transcriptional programs in a lineage- and state-dependent manner. This context selectivity is consistent with the notion that mechanotransduction outputs are determined by the integration of adhesion architecture, cytoskeletal tension, and baseline signaling circuitry 48-51. Mechanistically, one plausible model is that PC1, through its dual positioning within adhesion/ciliary signaling modules, modulates actomyosin tension and/or Ca²⁺-linked signaling nodes that impinge on Hippo kinase activity (e.g., MST/LATS), such that loss of PC1 can “release” TAZ activation only in cellular states where Hippo wiring is poised to respond to altered cytoskeletal/adhesion inputs. Consistent with this general principle, recent organoid-based studies have shown that force-dependent ECM remodeling can establish feed-forward mechanotransduction loops centered on YAP that drive invasion, emphasizing how baseline adhesion and ECM context can dictate downstream Hippo output 52. In addition, evidence for clinically relevant mechanotransduction modules (e.g., FAK-YAP signaling in lung cancer residual disease) supports the concept that distinct upstream mechanosensory architectures can engage Hippo outputs in a context-specific manner 53.

From a translational perspective, these data highlight PC1 as potential therapeutic target in solid tumors characterized by high mechanical responsiveness. Hampering PC1 mechanosensitivity may attenuate EMT, limit invasion, and modulate key mechanotransduction pathways such as TAZ signaling. Together with associations between PC1 expression and PD-L1 in lung cancer or PC2 expression and survival in HER2^+^ breast cancer, our findings raise the possibility that polycystins contribute to immune or targeted therapeutic resistance pathways. Future studies should explore combinatorial strategies integrating PC1 blockade with cytotoxic agents, immunotherapies, or mechanotherapeutic interventions targeting the stiffness of the TME. At the same time, translational extrapolation should be bounded by key uncertainties: the *in vivo* growth effects were modest, the clinical correlations are retrospective, and systemic interference with major mechanotransduction outputs can yield complex and sometimes unexpected consequences depending on tissue context and pathway dependency 54. Accordingly, defining “mechanically responsive” patient subsets, establishing predictive biomarkers (e.g., PC1/PC2-high with evidence of active mechanotransduction), and clarifying on-target liabilities will be important before considering PC1-directed mechanotherapeutic strategies, particularly given the broader field's experience that pathway-level Hippo/YAP/TAZ inhibition can have context-dependent therapeutic windows 55.

While this study provides multi-level evidence supporting a mechanoregulatory role for polycystins in solid tumors, several limitations warrant consideration. First, although our xenograft experiments demonstrated consistent suppression of EMT following PC1 inhibition, tumor growth reduction was modest, and the mechanistic basis of this discrepancy remains unclear. More detailed analyses of proliferation, apoptosis, stromal remodeling, and immune infiltration within the xenografts would strengthen conclusions regarding PC1's *in vivo* effects. Second, EMT characterization *in vitro* was limited to vimentin and β-catenin, and the observed context-dependent responses underscore the need for broader EMT and cytoskeletal profiling to capture lineage-specific programs. Third, TAZ regulation was assessed only at the level of activated protein; examinations of nuclear localization and downstream target genes would be necessary to establish direct mechanistic links between PC1 and Hippo pathway activity. Lastly, although our clinical datasets across four tumor types revealed robust correlations, retrospective cohort designs and limited sample sizes constrain generalizability, and functional validation of polycystin-associated clinical phenotypes remains incomplete.

In summary, our data support a model in which PC1-mediated mechanosensing represents a conserved upstream input across solid tumors, while the downstream transcriptional/phenotypic outputs are context-dependent, manifesting as relatively uniform EMT attenuation *in vivo* but divergent EMT-marker and Hippo/TAZ responses *in vitro* depending on lineage and baseline cell state. This conserved-versus-contextual framework helps reconcile the cross-tumor consistency of xenograft EMT effects with the cell-type selectivity of TAZ activation and underscores the value of stratifying mechanobiological dependencies by tumor context when considering polycystins as biomarkers or therapeutic targets.

## Conclusions

Collectively, our findings define polycystins, particularly PC1, as components of a tumor context-dependent mechanotransduction axis that links physical cues in the TME to epithelial-mesenchymal plasticity, migratory competence, and TAZ-mediated transcriptional regulation. The cross-tumor integration of human clinical correlations, *in vivo* functional modulation, and mechanotransduction effector analysis highlights the novelty of this work and established PC1 as a candidate biomarker and mechanobiological regulator in solid cancers. These results support further investigation of polycystin-mediated signaling as a potential point of therapeutic intervention in mechanically responsive tumors, an area of growing relevance for precision oncology 56, 57.

## Data Availability

The datasets used and analyzed during the current study are available from the corresponding author upon reasonable request.
